# Different Approaches for Manufacturing Ti-6Al-4V Alloy with Triply Periodic Minimal Surface Sheet-Based Structures by Electron Beam Melting

**DOI:** 10.3390/ma14174912

**Published:** 2021-08-29

**Authors:** Dmitriy Khrapov, Maria Kozadayeva, Kayrat Manabaev, Alexey Panin, William Sjöström, Andrey Koptyug, Tatiana Mishurova, Sergei Evsevleev, Dietmar Meinel, Giovanni Bruno, David Cheneler, Roman Surmenev, Maria Surmeneva

**Affiliations:** 1 Physical Materials Science and Composite Materials Centre, Research School of Chemistry & Applied Biomedical Sciences, National Research Tomsk Polytechnic University, 30 Lenina Avenue, 634050 Tomsk, Russia; dah8@tpu.ru (D.K.); mariakoz71@gmail.com (M.K.); mkk@tpu.ru (K.M.); rsurmenev@mail.ru (R.S.); 2 Institute of Strength Physics and Materials Science of Siberian Branch Russian Academy of Sciences (ISPMS SB RAS), pr. Akademicheskii 2/4, 634055 Tomsk, Russia; pav@ispms.tsc.ru; 3 SportsTech Research Center, Mid Sweden University, Akademigatan 1, SE-831 25 Östersund, Sweden; william.sjostrom@miun.se (W.S.); andrey.koptyug@miun.se (A.K.); 4 Bundesanstalt für Materialforschung und -Prüfung (BAM), Unter den Eichen 87, 12205 Berlin, Germany; tatiana.mishurova@bam.de (T.M.); sergei.evsevleev@bam.de (S.E.); dietmar.meinel@bam.de (D.M.); giovanni.bruno@bam.de (G.B.); 5 Institute of Physics and Astronomy, University of Potsdam, Karl-Liebknecht-Str. 24-25, 14476 Potsdam, Germany; 6 Engineering Department, Lancaster University, Lancaster LA1 4YW, UK; d.cheneler@lancaster.ac.uk

**Keywords:** Electron Beam Melting, scaffold, lightweight structures, computed tomography, Finite Element Analysis

## Abstract

Targeting biomedical applications, Triply Periodic Minimal Surface (TPMS) gyroid sheet-based structures were successfully manufactured for the first time by Electron Beam Melting in two different production Themes, i.e., inputting a zero (Wafer Theme) and a 200 µm (Melt Theme) wall thickness. Initial assumption was that in both cases, EBM manufacturing should yield the structures with similar mechanical properties as in a Wafer-mode, as wall thickness is determined by the minimal beam spot size of ca 200 µm. Their surface morphology, geometry, and mechanical properties were investigated by means of electron microscopy (SEM), X-ray Computed Tomography (XCT), and uniaxial tests (both compression and tension). Application of different manufacturing Themes resulted in specimens with different wall thicknesses while quasi-elastic gradients for different Themes was found to be of 1.5 GPa, similar to the elastic modulus of human cortical bone tissue. The specific energy absorption at 50% strain was also similar for the two types of structures. Finite element simulations were also conducted to qualitatively analyze the deformation process and the stress distribution under mechanical load. Simulations demonstrated that in the elastic regime wall, regions oriented parallel to the load are primarily affected by deformation. We could conclude that gyroids manufactured in Wafer and Melt Themes are equally effective in mimicking mechanical properties of the bones.

## 1. Introduction

The optimization of additively manufactured (AM) porous structures for biomedical applications aims at increasing fatigue life, enhancing mass transport properties for tissue regeneration, decreasing the occurrence of infections, minimizing powder release from the structures, and minimizing stress shielding. Stress shielding is caused by differences between Young’s moduli of the bone and the implant, and can be prevented by adjusting Young’s modulus of the implant through manipulating its structure (porosity) and material [1]. The designed porosity in regular-geometry lattice systems primarily depends on the type of unit cell. Usually, beam-based or sheet-based cell elements are used. AM porous structures based on the beam-like elements are intensively studied [2] and are most commonly used for porous scaffolds design.

Triply Periodic Minimal Surfaces (TPMS) have recently gained interest as the new approach to the design of the sheet-based porous scaffolds for tissue engineering. TPMS attracts attention due to zero-mean curvature at every point that is admittedly a great advantage since it improves the structure load-bearing capacity simultaneously assisting bone cell ingrowth [3]. The well-known TPMS are Schwarz Gyroid (G), Schwarz Primitive (P), and Schwarz Diamond (D) [4].

There are different approaches of using TPMS geometries for designing porous structures. First approach utilizes beam-based TPMS, strut-based [4], network-based or skeletal structure designs [5,6]. They are used to overcome stress concentrators in the sharp turns of the metal that are typical for unit cells with straight beam-like struts and a polyhedral core. Porous structures using straight beams can experience severe stress concentrations under loading, especially in regions where beams are merging, or bending at acute angles. Severe overloading and increased fatigue-related failure in the stress-concentration zones can lead to a complete collapse of the corresponding structural elements [7]. Beam-based TPMS are designed to have smooth struts and smoother connections between horizontal and vertical elements as compared to conventional beam-based structures. Most of the beam-based TPMS were manufactured using struts with circular cross-section. Beam-based TPMS structures were manufactured by the stereolithography rapid prototyping [8,9], selective laser sintering (SLS) [10] and laser powder bed fusion (L-PBF) [11]. Beam-based TPMS structures were also manufactured by Electron Beam Melting (EBM) by Yánez et al. [12,13] and Ataee et al. [14].

A second approach to designing TMPS structures is by using sheet-based [4] or sheet-like [15] elements. Such structures are also referred to as list structures or matrix phase lattices [5]. They comprise a wall of solid material bounded by two unconnected void regions. The continuity of the sheet-based TPMS is supposed to provide higher strength and damage-tolerance through effective obstruction of crack propagation [16]. Crack propagation in continuous sheet-based porous structures requires more energy as compared to common strut-based ones. Sheet-based gyroid structures also have higher Young’s modulus, peak stress, and toughness in comparison with beam-based gyroid structures. For example, Al-Ketan et al. [6] demonstrated that sheet-TPMS structures have superior mechanical properties in terms of Young’s modulus in comparison with conventional strut-based and skeletal-TPMS porous structures. Among such TPMS structures, gyroid structures attract the attention of many scientists. Kapfer et al. [17] demonstrated that the sheet-based gyroid structures have higher stiffness than the beam-based ones with the same porosity and manufactured from the same material. Aremu et al. [18] noted that gyroid lattices, unlike several other lattice types, possess axisymmetric stiffness making them desirable candidates for applications where the exact nature and direction of the loads are not fully known or if such loads are subject to large variations. Sheet-based structures have been manufactured by selective laser sintering (SLS) [6,15], L-PBF [5,19] but so far not by EBM. It is well-known that EBM structures have lower resulting porosity [18] and lower residual stress as compared to similar L-PBF- and SLS-manufactured structures because of the preheating during manufacturing that acts as a stress relief heat treatment [20,21]. Internal defects of the EBM-manufactured objects affect their fatigue life [22]. To evaluate manufacturing quality and porosity, the size and the form of the defects process monitoring approaches for is investigated [23]. Moreover, manufacturing parameters’ optimization leads to porosity reduction [24]. One way to optimize manufacturing parameters is EBM process simulation that helps to predict physical properties of AM [25,26]. To evaluate the behavior of the Ti-6Al-4V implant in human body, friction and wear performance of the wrought and EBM-manufactured Ti-6Al-4V in simulated body fluid solution were studied [27].

Studying EBM TPMS sheet structures is of great interest for biomedical applications as the manufacturing of implants with porous elements is one of the core applications of EBM technology. Sheet-based TPMS can be produced by EBM using different manufacturing parameter sets commonly known as Themes. “Melt” Themes originally designed for manufacturing solid structural elements require a 3D model with predetermined material thickness. The “Wafer” Theme originally designed for manufacturing different support structures uses zero-thickness 3D models [28]. Taking into consideration the complexity of TPMS, the Wafer Theme may become a new key to controlling specimen’s porosity and preventing the stress shielding effect. The differences between the gyroid samples manufactured using Melt and Wafer Themes are the subject of this work.

Our initial assumption was that specimens based on 200 µm thick model manufactured using Melt Theme and specimens based on zero-thickness model manufactured using Wafer Theme would have identical sheet thickness and identical mechanical properties. This assumption was based on the fact that the beam spot diameter set in the ARCAM A2 machine is commonly 200 µm [28].

In this work, we investigate the mechanical properties of the TPMS porous specimens based on model with 200 µm thickness manufactured by EBM using the Melt Theme and specimens with an equivalent design but produced by EBM using the Wafer Theme, zero-thickness model. Samples were manufactured using the standard Themes provided by ARCAM EBM for Ti-6Al-4V alloy.

We address the relationship between structural performance and manufacturing modality, keeping porosity constant. The novelty of the research lies in combination of design methods of TPMS and EBM-manufacturing modalities. The aim of the current investigation was to evaluate the worthiness of Wafer Theme in comparison with the Melt Theme for TPMS structures’ fabrication from the mechanical point of view.

## 2. Materials and Methods

### 2.1. TPMS File Preparation

Wolfram Mathematica (version 12) [29] was used to visualize the gyroids using the following equation:

(1) sin(kx)cos(ky)+sin(ky)cos(kz)+sin(kz)cos(kx)=0

**Figure 1:**
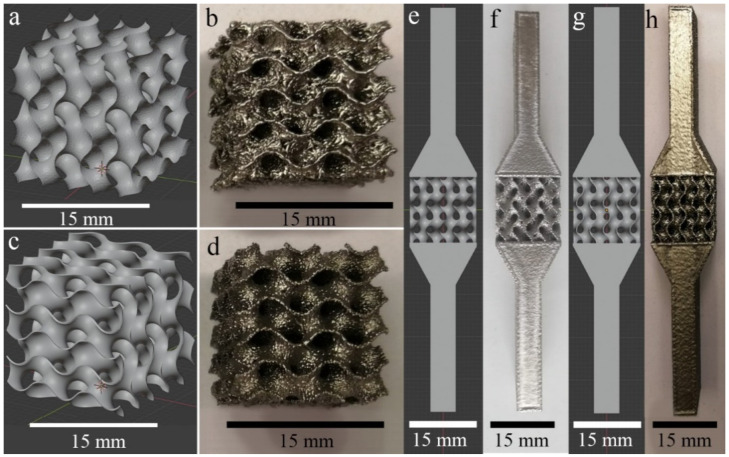
Samples for compression tests: (**a**) 3D model with zero thickness; (**b**) WT specimen; (**c**) 3D model with 200 µm thickness; (**d**) MT specimen. Samples for tension tests: (**e**) 3D model with zero thickness; (**f**) WT specimen; (**g**) 3D model with 200 µm thickness; (**h**) MT specimen.

The limits of the surface were chosen from −5/2 π to 5/2 π in all directions. So, the designed structure consists of three unit cells along each coordinate direction (X, Y, Z), resulting in a total size of 15 mm × 15 mm × 15 mm. The coefficient *k* controls the unit cell size. For this research *k* = 1 was chosen. Two different sets of the 3D models were implemented. The models of the first set with zero-thickness were exported from Mathematica as STL files with the default density of the polygon mesh, Figure 1a. 

The second set of models was produced from the first one by assigning a thickness of 200 µm to all surfaces, Figure 1c and also was exported from Mathematica as STL files with the same conditions. Since the surface of gyroid consists of semicircular surfaces, the exported 3D models had a large number of vertices (more than 10^6^), thus creating a high-poly model. The design files of large size can in some cases cause memory issues of ARCAM Build Assembler software. Thus, the high-poly meshes generated with random polygon size and shape distribution required additional mesh optimization. For this purpose, the MeshLab software, an open-source Mesh Processing Tool [30], was used. The number of the vertices was steeply reduced to 10,000 for zero-thickness model and to 44,000 for 200 µm thick model, preserving the boundaries and the topology of the mesh. Topological errors, such as non-manifold faces, self-intersections, duplicate faces, etc., were also removed using the MeshLab. 

For the tensile samples, two opposite 4 cm long tapering blocks were added to the gyroid structures of both types, Figure 1e,g. The total length of these specimens was 95 mm. The process of error correction was repeated in FreeCAD [31]. This software was used for STL to STEP file conversion (necessary for the FE analyses). The obtained STL files were used for designing all specimen models and for processing in the ARCAM Build Assembler—for EBM—manufacturing.

### 2.2. Finite Element Analysis

The aim of FE simulations was to qualitatively analyze the deformation process and the stress distribution. The models with thicknesses of 0.25 and 0.4 mm were imported to ANSYS Workbench (ANSYS, Canonsburg, PA, USA). The values of thicknesses were taken based on the experimental results (see Section 3.2). A tetrahedral mesh model was implemented. This method was convenient since the initial models obtained in Wolfram Mathematica consisted of triangle polygons. The total number of nodes and elements for the model with the thickness of 0.25 mm were 910,727 and 489,256, respectively. Using the physical properties of Ti-6Al-4V, the modeled specimen had an estimated mass of 1.53 g and a porosity of 85%. The total number of nodes and elements for the model with the thickness of 0.4 mm were 1,070,675 and 610,874, respectively. Using the physical properties of Ti-6Al-4V, the modeled specimen had an estimated mass of 3.18 g and a porosity of 75%. The von Mises failure criterion was chosen, and a yield strength of 970 MPa for Ti-6Al-4V was selected [32]. Boundary conditions were applied as follows: frictionless support was applied to the bottom face and 1 mm displacement was applied to the top. Only the elastic regime was simulated for both tension and compression tests.

### 2.3. Manufacturing

Scaffolds were manufactured from Ti-6Al-4V powder in ARCAM A2 EBM machine by ARCAM, EBM (Mölnlycke, Sweden) operating under firmware version 3.2 using standard Themes without modification of the default parameter settings. All samples were manufactured using 50 μm layer thickness and process temperature of 720 °C. 

In beam-based structures, PBF AM specimens can be manufactured using different beam energy application strategies, influencing, in particular, melt pool dimensions, solidification rate and final material microstructure. Modern PBF AM machines are commonly operated using parameter sets provided by the manufacturer. An ARCAM “Theme” is a set of settings incorporating beam scanning strategy and process parameters. The standard Themes provided by the EBM machine manufacturer ARCAM EBM are called “Melt”, “Net”, and “Wafer”. The “Melt” Theme is optimized for manufacturing of solid structural elements. The “Net” Theme is designed for the manufacturing of the beam-based porous structures. The “Wafer” Theme is designed for manufacturing of support structures (essential elements of the EBM process), where wafer-thin surfaces are required [28]. These supports are commonly used for stabilizing overhanging elements, and as spacers between the solid elements and the base plate. Such supports are removed after manufacturing. So, the “Wafer” Theme was designed to produce very thin structures to guarantee a small amount of waste material. Structures manufactured using Net and Wafer Themes are designed as zero-thickness geometry, and the cross-section of the elements is defined by the beam energy and its deposition rate.

The “Melt” Theme is more complicated than the “Wafer” one. The first path of the beam (called first contour) is shifted out from the CAD-defined element periphery by a certain value called the first contour offset (CO1). Next, the beam performs a second contour scan with a second contour offset (CO2) moving slightly inward from the CAD-defined cross-section periphery. After that, the beam melts the area enclosed by these two contours (hatching) using different types and strategies of the raster motion. Corresponding contours are performed by continuous beam spot motion over the whole periphery length, or in so-called ‘Multibeam^TM^ mode’. In the latter case, the beam moves only through the short sector of the contour and ‘jumps’ away to melt another sector, repeating the operation many times to cover all needed contours. The main purpose for selecting single- or double- contours in continuous or Multibeam^TM^ mode is the optimization of the process to obtain the smallest possible roughness of side surfaces of the components. It is clear that when using any mode for the manufacturing of lightweight and porous structures, careful parameter selection is needed to guarantee that the resulting element cross-sections are as close to the CAD design as possible. When the elements with a small cross-sectional area are EBM-manufactured using Melt Theme, automated file preprocessing can reduce the number of contours (in an automatic fashion) until only the hatch is left, minimizing the increase in the dimensions of the manufactured elements.

The “Wafer” Theme, traditionally, is mainly used for the plane sheet supports with relatively small surface areas; our experience shows that it is also effective when used for quite complicated structures. Slicer software created a line pattern for WT based on zero-thickness gyroid model for each slice, while for MT, it created areas for melting based on 200 µm thickness gyroid model. Depending on the parameter settings, thin structures made with Wafer Theme can have a certain amount of through-holes. However, it is clear that application of the Wafer Theme for EBM of sheet-based lightweight and porous structures can bring significant improvement of the mechanical properties of the resulting components.

Specimens manufactured using Melt Theme and Wafer Theme are further referred to as MT and WT correspondingly. Examples of design structures and manufactured EBM WT and MT samples are presented in Figure 1.

In the build file, WT samples were placed directly on the base plate and were oriented with the side walls normal to the build direction. MT models were oriented in such a way that their cross-section diagonal was aligned with the build direction. 

After manufacturing, the specimens were removed from the powder bed and separated from the base plate in a standard ARCAM Powder Recovery System. All specimens were subjected to compressed airflow for more than 10 min, thereby ensuring the removal of the powder particles loosely connected to the surfaces.

WT specimens were manufactured with turned off Multibeam^TM^ contour mode (continuous beam path), beam current of I = 5 mA, and scanning velocity of v = 1000 mm/s. Three specimens were manufactured for each of tension and compression tests. The fixation heads for all tension specimens were manufactured using Melt Theme with default parameter settings, Figure 1f, h. Equivalent parameters of the manufactured compression specimens are given in Table 1. 

The surface morphology was studied by scanning electron microscopy Quanta 200 3D (FEI, Eindhoven, The Netherlands). 

The outer dimensions of the fabricated scaffold samples were measured by a Vernier caliper; all specimens were weighted on an Acculab ALC-210d4 (Sartorius AG, Göttingen, Germany) scale. Their calculated solid volume and measured weight were used to determine the apparent density *ρ* of the structure (presented in Table 1). Assuming the density of solid Ti-6Al-4V is equal to *ρ*_0 =_ 4.43 g/cm^3^, the porosity *P* of the scaffolds in % was obtained by:

(2) $$P=1-\frac{\rho }{\rho_{0}}$$

### 2.4. X-ray Computed Tomography

The X-ray computed tomography (XCT) measurements were performed at BAM using an XCT scanner, developed together with the company Sauerwein Systemtechnik (today RayScan Technologies GmbH, Meersburg, Germany). A microfocus X-ray tube XWT-225-SE (maximum voltage 225 kV) from X-RAY WorX GmbH (Garbsen, Germany) was used as a source. An XRD1620 (CsI scintillator, 2048 × 2048 pixel) detector from PerkinElmer Inc. (Waltham, MA, USA), with in-house built enclosure and cooling system was used. A tube voltage of 120 kV and a tube current of 120 μA were used during the scans. The voxel size was 15.3 µm. The reconstruction of 3D volumes from 2D projections was made by the software developed in BAM using a filtered back-projection algorithm [33]. The obtained raw data files were analyzed using VGStudio MAX 3.3 by Volume Graphics, Heidelberg, Germany. The STL files of the 3D models and the XCT-based reconstructions were used to conduct Nominal/Actual comparison. The wall thickness was evaluated by the sphere method. 

### 2.5. Mechanical Tests

Quasi-static uniaxial compression and tension tests were performed using a universal testing machine INSTRON 3369 and INSTRON 5582, (Instron Deutschland GmbH, Darmstadt, Germany), with a 50 kN load cell. Tests were conducted at 20 °C according to ISO 13314:2011 [34], and using a displacement rate of 0.5 mm/min. Through the measurement of the applied load, we calculated the stress dividing the load by the effective area of the lattice structures. The failure strain was set at 50% of the specimen height. Results for the quasi-elastic gradient [35], compressive offset stress, first maximum compressive strength, energy absorption at 50% strain (Equation (3)), and specific energy absorption (Equation (4)) were calculated following ISO 1331 standard [34].

The ISO 13314 was devoted to describing the mechanical behavior of beam-based structures. TPMS sheet-based scaffolds represent structures with more complex shapes than beam-based structures. Indeed, the term “unit cell” is obvious for beam-based structures, but not for sheet-based structure. We therefore took ISO 13314 as a reference at the stage of designing the scaffold but did not strictly follow it. In fact, our goal was to qualitatively assess the behavior of our scaffolds, not to qualify them for production. Indeed, there are many scientific articles not strictly following the requirements of standard test or production methods [5,35,36,37,38]. 

The quasi-elastic gradient E_qe_ [4,39,40,41,42,43] of the porous samples is the gradient of the straight line determined within the linear deformation region at the beginning of the compressive stress-strain curve, i.e., this value is defined similarly to Young’s modulus E for bulk material. Additionally, the compressive offset stress and the first maximum compressive strength for porous specimens are defined similarly to the yield stress *σ_y_* and the compressive strength *σ_max_* for bulk specimens. Yield strain was defined as 0.2% strain, and the compressive offset stress was determined accordingly. Quasi-elastic gradient *E_qe_*, yield stress *σ_y_*, and ultimate tensile strength *σ_max_* were estimated for tensile specimens. The plateau stress *σ_pl_* is the arithmetical mean of the stress values between 20% and 40% compressive strain. The point in the stress-strain curve at which the stress is 1.3 times the plateau stress is defined as the plateau end. It can be used for the determination of energy absorption and energy absorption efficiency:

(3) $$W=\frac{1}{100}\overset{e_{0}}{\underset{0}{\int }}\sigma de$$

where *W* is energy absorption per unit volume (MJ/m^3^), *σ* is the compressive stress (MPa), *e*_0_ is the upper limit of the compressive strain. The energy absorption per unit volume was calculated from the area under the stress-strain curve up to 50% strain.

The crashworthiness of a material can be expressed in terms of its specific energy absorption. The specific energy absorption (Equation (3)) *ψ* is defined as the work *W* performed per unit weight when the material is compressed in a uniaxial manner up to a specific strain. The strain of 50% [41,44] was chosen for the evaluation of specific energy absorption:

(4) $$\psi =\frac{W}{\rho };W=\overset{\varepsilon }{\underset{0}{\int }}\sigma d\varepsilon$$

where *ρ* denotes the mass density, *σ* the axial stress, and *ε* the work-conjugate axial strain [45].

## 3. Results

### 3.1. Scanning Electron Microscopy

**Figure 2:**
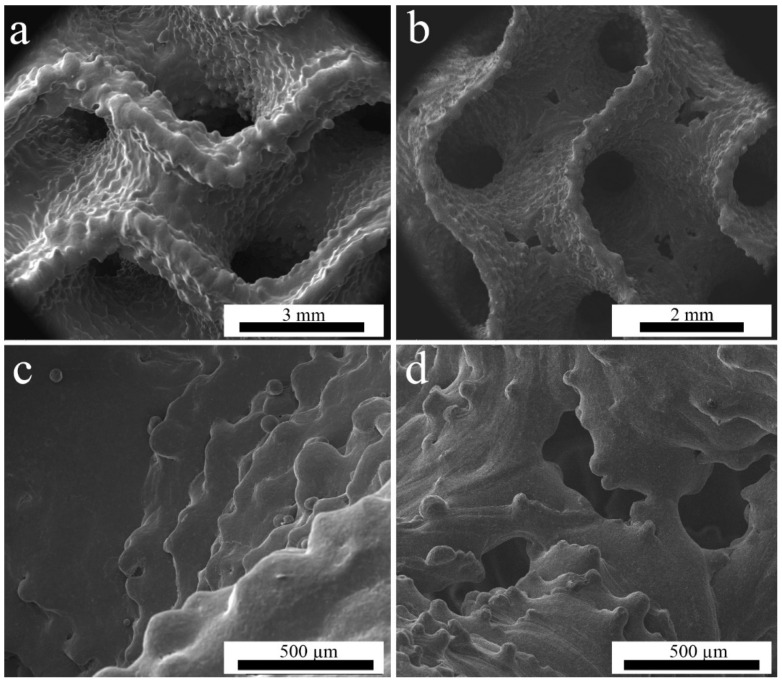
SEM images of (**a**,**c**)—MT specimen; (**b**,**d**)—WT specimen.

The gyroid structures present both partially melted powder particles on the surfaces (Figure 2a,b) and the stair effect (Figure 2c,d), typically observed in AM parts (due to the layer-wise manufacturing) [36,37,38,39,40,41,42,43,44,45,46,47,48]. While the minimum electron beam spot size of EBM machine is about 200 µm, the melt pool is commonly wider, and the smallest possible sheet thickness that can be resolved in EBM is about 200 µm [28]. The wall thickness of the WT specimens was smaller than that of MT, as can be observed in Figure 2a,b. 

WT structures have quite small sheet thickness, as the beam energy used to manufacture them is rather low. The production of such thin structures is at the limits of the machine capabilities, since the powder particle size is between 75 and 125 µm. Consequently, some holes were present in the sheets (Figure 2b,d). Thicker sheets of the MT structures have visibly higher roughness but no through-holes. WT samples have the holes predominantly in the areas where the surface is parallel to the build plane. This is well-known in PBF techniques, where such problems occur in overhanging elements and thin structures normal to the build direction [48].

### 3.2. X-ray Computed Tomography 

**Figure 3:**
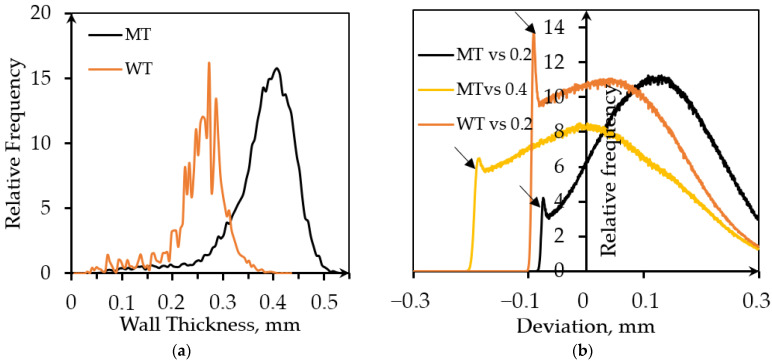
Evaluation of the parameters based on the XCT data analysis: (**a**) Wall thickness distribution; (**b**) Deviation distribution obtained from Nominal/Actual Comparison analysis.

XCT was used to describe inner structure, evaluate wall thickness, and reveal the difference between the designed structure and the manufactured specimen. The wall thickness of WT and MT EBM structures evaluated from XCT reconstructions is presented in Table 2. The wall thickness distribution in the two specimens is presented in Figure 3a. Note that for both specimens, the targeted wall thickness was 0.2 mm. The actual mean wall thickness was about 0.4 mm and 0.25 mm for MT specimen (bulk-melt mode with contours) and WT specimen (Wafer-mode), respectively. It is clear that for thin-walled structures, the contour-enabled bulk melt mode is not optimal and leads to a larger than desired wall thickness.

The reason for the difference between thicknesses of the designed and MT manufactured structures lies in additional thickness caused by two contours and, probably, wider than expected melt pool. Since the surface of the gyroid is curved, it is impossible to evaluate roughness by traditional methods. It is known that arithmetic roughness (R_a_) for vertical struts of the EBM-manufactured structures is about 40 µm, while the mean value of the maximum height of the surface profiles of vertical struts (R_t_) is 212 µm [28]. Comparison of a designed 3D model and as-manufactured samples performed with the standard VGStudio function named ‘Nominal/Actual Comparison’ characterizes the manufacturing accuracy and supplements the wall thickness analysis, Figure 3b. Nominal/Actual Comparison is also an alternative way to describe roughness of the manufactured specimens in qualitative terms. The value of average surface roughness is comparable with the sheet thickness that is quite typical for EBM-manufactured porous structures [49].

It makes no sense comparing zero-thickness model used for designing WT samples and 0.25 mm thick reconstruction. Therefore, a model with the desired thickness was used for the Nominal/Actual Comparison. The average wall thickness of the MT specimen based on the 0.2 mm model was about 0.4 mm. A comparison with initial model and, additionally, with 0.4 mm thick model was performed to evaluate surface roughness more precisely.

The deviation of the structure was estimated from both sides of the walls. The searching distance was 0.3 mm, which is quite large in comparison with the average wall thickness. The positive deviation is caused by the presence of contours in MT samples, melt pool ‘swelling’ into the surrounding powder bed, and the presence of powder particles partially merged with the surface. There are sharp peaks of the relative frequency at the negative deviations followed by rapid drops (indicated by arrows). They may be attributed to the surface elements detected from the opposite side of the wall, and this will be typical for porous lattices with any unit cell design.

**Figure 4:**
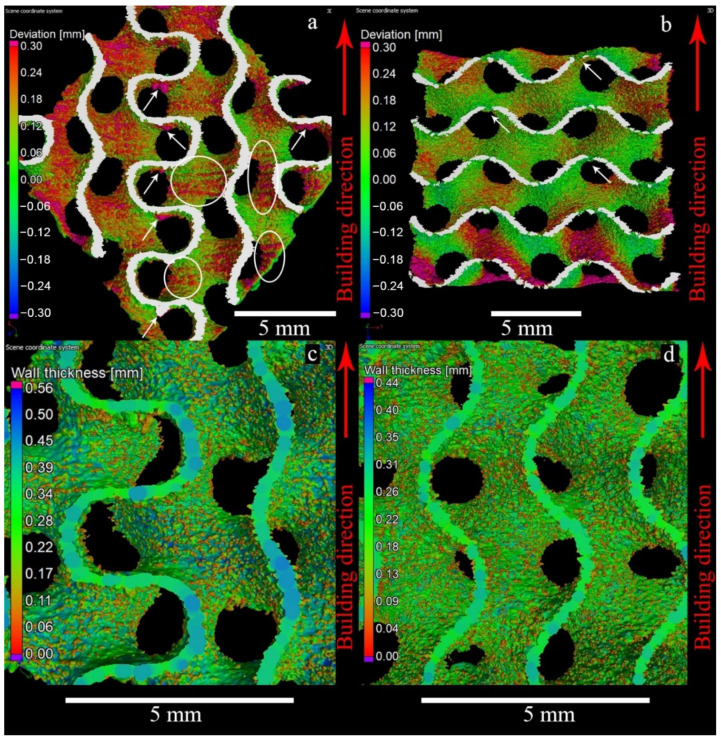
3D rendering of the CT reconstructions for the Nominal/Actual Comparison of the MT gyroid (**a**) and WT gyroid (**b**). Visualization of the wall thickness for the MT gyroid (**c**) and WT gyroid (**d**). White arrows indicate stalactite-like structures on the horizontally oriented parts of the MT walls (areas parallel to the layers, purple color). The white circles highlight the partially melted powder particles attached to the surface of the vertical areas.

Figure 4 illustrates the Nominal/Actual analysis of the samples. White arrows in Figure 4a indicate stalactite-like structures on the horizontally oriented parts of the MT walls (areas parallel to the layers, purple color). This effect is quite common in the PBF- manufactured specimens [50]. Interestingly, it is much less pronounced for the WT specimens due to the smaller beam energy used. In the MT samples, clusters of the partially fused powder particles are also present. The white circles highlight the partially melted powder particles attached to the surface of the vertical areas. This effect was not found for WT specimens, presumably because of the smaller electron beam energy input, Figure 4b. The purple area on the lower part of WT specimen indicates larger dimensional deviation in the first layers. This is a known effect due to the incompletely stabilized temperatures in the semi-sintered powder surrounding the melt pool at the early stages of the build, and uneven compensation of the expansion of powder placed under the start plate. Uneven compensation of the powder expansion leads to the situation when during some of the first layers, EBM rake brings no powder for some parts of the working area. Moreover, sample material starts to be deposited only after a few nominal layers leading to the distortions of the samples that are due to start from the base plate. Additionally, the material adjacent to the start plate can be distorted and have some different microstructure due to diffusion of metal ions from base plate [28]. 

**Figure 5:**
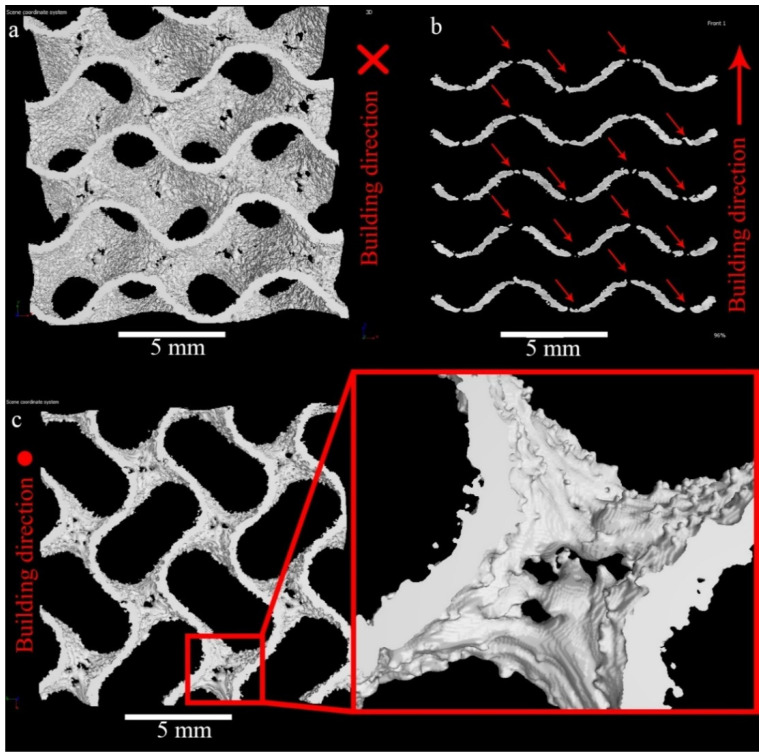
3D rendering of CT-reconstruction of WT gyroid vertical (**a**,**c**) and horizontal (**b**) views (**a**—bottom view; **c**—top view; **b**—side view).

The through-holes are present nearly in each surface of WT samples parallel to base plate, Figure 5. The shapes of the pores are irregular; in some cases, holes are interconnected, Figure 5a,c. Red arrows in Figure 5b indicate the holes visible in the vertical cross-section of the sample. Careful investigation of the wall surfaces shows the tendency of the up-facing walls, Figure 5c, to have lower roughness than the down-facing ones, Figure 5a. 

### 3.3. Mechanical Properties

#### 3.3.1. Compression Tests

**Figure 6:**
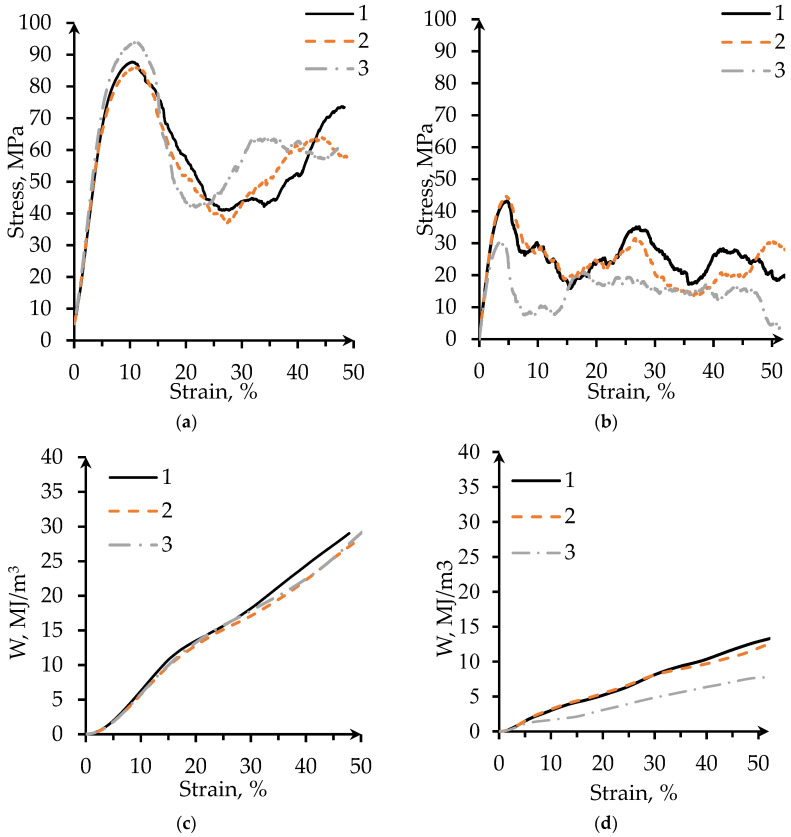
Compressive stress-strain curves for the gyroid samples manufactured in (**a**) MT; (**b**) WT. (Note the different y-axis scales). Energy absorption per unit volume versus strain curves for lattice samples of (**c**) MT; (**d**) WT. (Note the different y-axis scales).

Stress and energy absorption vs. strain curves for the compression samples are presented in Figure 6. Characteristics of such curves for the WT samples show a larger scatter than those for the MT ones. Most probably this can be explained by the statistical variation of the shape of the hole-type defects in the WT structures (Figure 2a,b), but the intrinsic variation of the wall thickness also contributes to the scatter. The compressive strength, for instance, can vary by over 30% among samples. The first maximum compressive strength of the WT samples is about twice lower than that of MT samples. 

Conventional cellular structures with uniform density exhibit three deformation regimes during compressive testing [46]: a linear elastic compression, a plateau with approximately constant stress, and a final densification with steeply increasing stress. Sheet-based TPMS structures except gyroid ones are reported to have fluctuations of the curve in the plateau regime [4,6]. The mechanical behavior of our specimens does not fully follow this description. For MT specimens, a single drop and recovery in the strength in the plateau region can be observed, Figure 6a,b. The stress-strain curve of the WT shows a drop in the strength of the structure right after the peak strength is reached. This may be a result of sudden fracture of the wall element in one layer. The following fluctuations in the plateau region can be observed. According to Al-Ketan et al. [6], the fluctuations in strength can take place due to collapse or fracturing events of cell layers, while the recovery is due to local densification of the collapsed layer where the load is transmitted directly to the next layer of cells. The values of the plateau stress were calculated based on the ISO 13314 [34] (see Section 2.5). The plateau stress of WT gyroid is about 15 MPa, whereas it is about 49 MPa for MT gyroids. 

**Figure 7:**
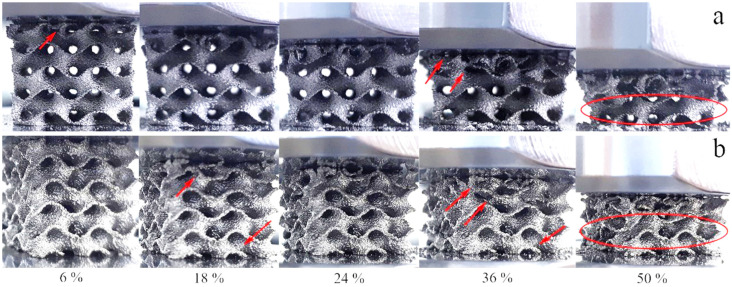
Steps of mechanical deformation during compression: (**a**) WT; (**b**) MT. Red arrows indicate places where the structure lost integrity during compression. The encircled layers keep integrity even at the 50% strain.

Figure 7 presents photographs of the WT and MT structures at different deformation stages, corresponding to 6%, 18%, 24%, 36%, and 50% of the overall strain. Both WT and MT samples mainly display layer-by-layer deformation behavior. Plastic deformation is also visible in both sample types. Such deformation is more evident in the vicinity of the collapsing layers.

After the first maximum of compressive strength, a deep fall of the stress can be observed (Figure 6a,b). For the MT structure, this fall starts around 15% strain and corresponds to the collapse of the first layer (Figure 7b). The WT structures display several stress maxima and minima because the individual cell walls come into contact with each other after collapsing of a layer. In this case, the first minimum around 7–15% strain corresponds to the collapse of the first layer (Figure 7a). At larger strains, the stress grows again; this region is described by some authors as strain-hardening [46]. The structure becomes stronger because of the densification of the crushed layer. While losing its dimensions, the specimens recover a part of the initial crushing strength before reaching 50% strain [5]. 

It is worth mentioning that WT specimens reach first maximum compression stress at 5% of strain, whereas MT specimens reach it at 10%. The yield strain, the compressive offset stress, and the first maximum compressive strength of the MT specimen are twice as large as that for WT specimen. Specific energy absorption at 30% strain for MT porous structure is 1.5 times higher than that for WT structure, see Figure 6c,d and Table 3. However, specific energy absorption at 50% strain for the two structures is quite similar.

#### 3.3.2. Tensile Tests

**Figure 8:**
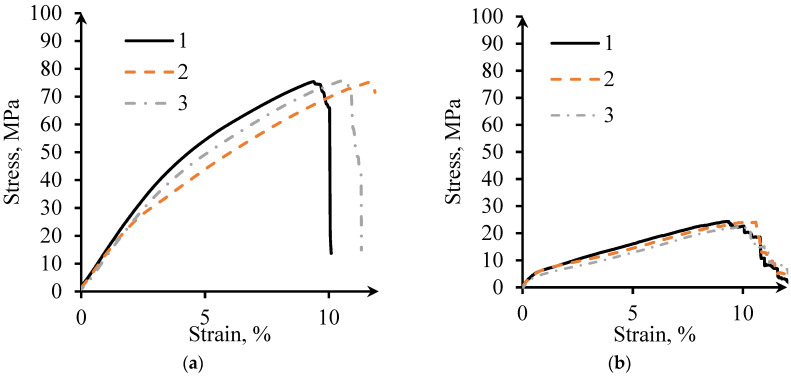
Tension stress-strain curves: (**a**) MT; (**b**) WT.

Figure 8 shows the tensile stress-strain curves for MT and WT specimens. The tensile behavior of the samples manufactured using different Themes are similar. Ultimate tension stress (UTS) of the MT specimens is three times higher than that for WT specimens; however, their quasi-elastic gradients for both themes are equal to 1.2 GPa. The tensile failure strain of this type of specimen is about 10%.

### 3.4. Finite Element Analysis

**Figure 9:**
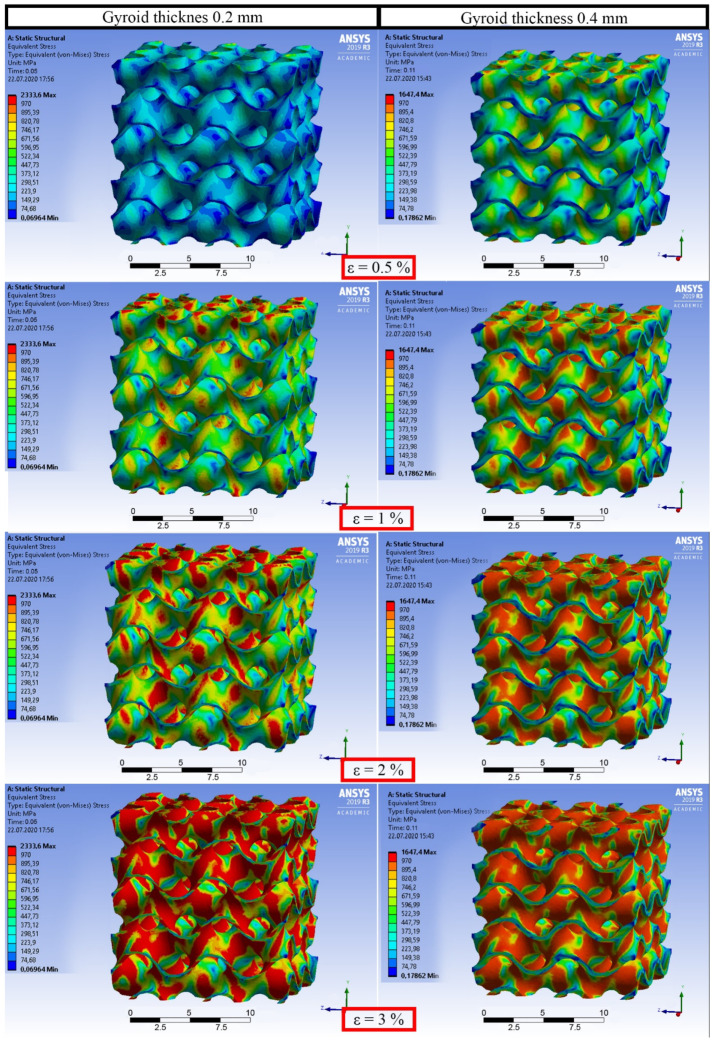
Simulations of the stress distribution during uniaxial tension (elastic region) of the gyroids with different thicknesses.

To analyze the elastic deformation process and the stress distribution during tensile tests, FE simulations were employed. The simulated stress distributions in the elastic region of a tensile test of both MT and WT models are displayed in Figure 9. The simulated stress distributions during a compression test (elastic range) were similar to tensile ones and are not presented here. In this research, a fine mesh has been used and the dependency of the results to the mesh size has been checked.

Interestingly, during elastic deformation of the structure, only the vertical elements of the gyroid wall are affected by mechanical deformation. These elements together have spiral shapes due to the specific design of the gyroid. They are stress-sensitive and reminiscent of elastic springs. This phenomenon matches the description of inclination angles influencing the manufacturability of TPMS described by Yang et al. [38]. As reported, the most frequent surface orientation within the experimental gyroid structure had an inclination angle of 55° to the build plane, while the least frequent possess around 0° inclination angle. The yielding process of the metal initiates in the vertical areas located parallel to the load direction and more actively continues in the diagonally (55°) oriented parts. The yielding process finally reaches horizontally aligned saddle points. Therefore, the holes appeared in these areas because of tessellation discrepancy influence on the mechanical behavior only after the whole specimen reaches the yield strain, and they do not affect the quasi-elastic gradient.

## 4. Discussion

The investigation results show that the sheet-based gyroid structures present an interesting combination of morphological properties and quasi-static mechanical behavior. Thin-wall gyroid structures obtained in EBM-manufacturing using different Themes were expected to have identical geometrical and mechanical properties due to initial settings. In the current research, the complex shape of the final gyroid samples was largely affected by the choice of the Themes, and fused powder particle distribution influencing the surface roughness and effective wall thickness. Such rough beaded surfaces are typical for AM lattice structures. Suard et al. investigated the influence of the fabrication angle on the surface roughness of EBM struts [49]. They reported that for a vertical strut, the roughness does not significantly fluctuate, while the roughness of oblique and horizontal struts increases on the down-facing surfaces. This was assumed to be due to the partial over-melting and reduced cooling, which lead to the ‘leakage’ of the melt and formation of the stalactite-like micro-columns and blobs (in our case indicated by white arrows in Figure 4a). It was established that for significant overhanging angles (more than 45° from horizontal), a greater number of partially molten powder particles are attached to the downward surfaces [51]. Moreover, Yang et al. demonstrated that sheet-based Ti-6Al-4V gyroids manufactured by LPBF have different surface morphology between up- and down-facing surface areas [38].

For the MT specimens, the beads are formed mainly on surfaces parallel to the building direction. Thus, the vertical walls had effective thickness up to 0.5 mm, while the oblique and horizontal walls had a thickness of 0.25 mm, Figure 4c. Typically, in the beam-like structures, there is an increase of the surface irregularities in struts produced by EBM with direction diverging from vertical [49]. However, in sheet-based structures, vertical walls are thicker, as it seems that the contours of the Melt Theme strongly influences resulting thickness of the oblique and horizontal walls. WT specimens possess quite uniform thickness for oblique and vertical wall areas, Figure 4d. Since holes are systematically present in the horizontal walls, no thickening in the WT was observed.

For porous samples, three types of compression failure are known [5]. The first type is layer-by-layer failure. This type is characterized by the collapse of cells in planes perpendicular to the manufacturing and loading direction: each layer collapses into the one below. The second type is brittle fracture of the cell walls and propagation of cracks through the lattice. Commonly, the fracture starts at a pre-existing defect, such as an internal pore of a surface irregularity. A crack can fork perpendicularly to its direction of propagation through the walls of the cells, implying that crack propagation through the structure is likely tortuous. The third type is diagonal shear. In some cases, combined diagonal shear and layer-by-layer failure occurs, for example, for sheet-based gyroid manufactured by SLS from maraging steel [6]. The compressive failure mode of the gyroid lattice is related to the size of its constituent cells.

The different stress values of the MT and WT specimens can be explained by the different relative density, while the different shape of the stress-strain curves is most probably due to the influence of the through-holes and smaller wall thickness in the WT samples, favoring Euler instability of the walls under compressive load. Such instability has a statistical character, as it occurs in single walls separately (or in groups of walls favorably oriented with respect to the load axis).

Interestingly, compression tests revealed that despite the difference in wall thickness and material volume, the two types of specimens have similar quasi-elastic gradient (around 1.5 GPa). Following a Gibson-Ashby law [52,53]:

(5) $$E=E_{0}\times {\left(1-P\right)}^{n}$$

One can calculate the shape factor *n* using the known porosity values *P* and the Young’s modulus of Ti6Al4V (*E*_0_ = 110 GPa). Corresponding values are *n* ~ 2.3 for WT and *n* ~ 3.2 for MT samples. Since *n* = 2 corresponds to a cellular structure of compacted overlapping pores and *n* = 3 to a cellular structure of compacted non-overlapping pores [52], we can observe that thinning the walls not only causes an increase of porosity but also makes elastic behavior of the samples more similar to a strut-like cellular structure. The MT structure even tends to the elastic behavior of a cellular structure of overlapping spheres (*n* = 4).

Importantly, the values of the quasi-elastic gradient satisfy the limits of the elastic moduli for human cortical bone [54]. Moreover, the specific energy absorption at 50% strain for the two structures is quite similar, showing that EBM WT structures are as strain tolerant as MT.

It was demonstrated by Yang et al. that L-PBF Ti-6Al-4V gyroid sheet-based structures under compression load behave as bending-dominated structures [38], while Al-Ketan et al. [6] and Kelly et al. [35] showed stretching dominated deformation for the sheet-based gyroids made of maraging steel and Ti-6Al-4V, respectively. The linear elastic regime is driven by the bending of the inclined cell walls or by the stretching of the vertical cell walls.

Mechanical properties such as compressive offset stress, yield strain, first maximum compressive strength and energy absorption are different for sheet-based gyroid manufactured in Melt Theme and Wafer Theme. However, the quasi-elastic gradients and specific energy absorption at 50% strain of these structures are equal. Thus, WT structures are as strain tolerant as MT ones.

Comparison of the quasi-elastic gradient values (in compression) of the studied gyroid sheet-based samples to the ones reported in the literature are presented in Table 4.

L-PBF-manufactured sheet-based gyroids have quasi-elastic gradient significantly larger than EBM-manufactured ones. This can be attributed to surface roughness of the EBM-produced specimens. The highest value of quasi-elastic gradient in the Table 4 belongs to the specimen with the 50% porosity.

Thin walls allow decreasing the distance between neighboring walls, keeping the ability to effectively remove powder from the structure. In fact, it is essential that porous samples used for the mechanical and flow tests be very thoroughly cleaned from residual powder. The semi-sintered powder remaining inside the structure would influence its permeability and mechanical behavior. Additionally, thorough cleaning is critical for the applications where even slow release of loose powder during the component life is completely unacceptable, as in the case of implants. All TPMS structures used in this study were successfully cleaned with conventional powder recovery system (see e.g., [55]).

**Figure 10:**
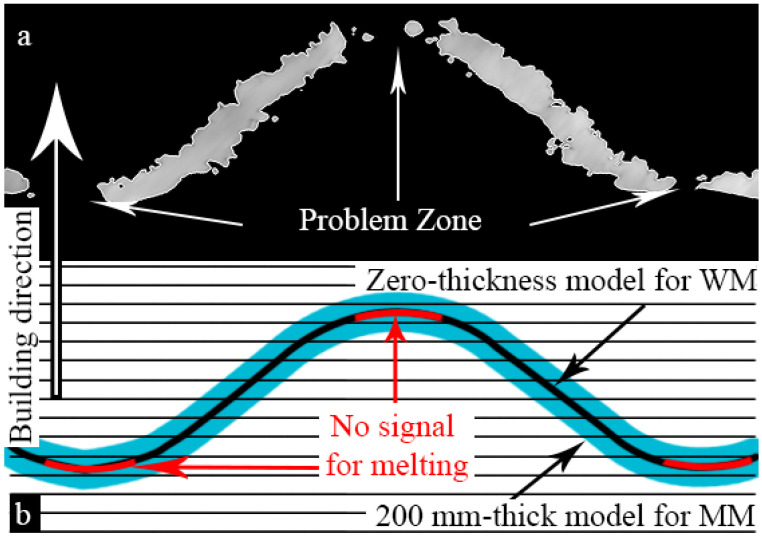
Assumption on the through-holes’ appearance: (**a**) CT in vertical cross-sectional view; (**b**) Scheme of manufacturing process in vertical cross-sectional view.

Using FE simulation, it was shown that relatively small number of through-holes occurring in thin-walled structures does not influence the elastic behavior of the lattice. In case of the WT structures, through-holes are wider and more abundant in the surface sections having small inclination to the layer plane (Figure 10). Two reasons could be responsible for that: first one, related to technology, and second one, to the design process. Technologically, overhanging elements without supports are always problematic in PBF AM. From the point of view of design, since the 3D model (CAD) for WT samples has zero thickness, the part of the STL file corresponding to this problem area cannot be registered by slicer software. The tangent point represents, therefore, a blind spot, where the electron beam could be turned off (Figure 10a). Further studies into this phenomenon are needed, and corresponding corrections of the zero-thickness design model should be incorporated in the future.

It is interesting that the MT specimens, with porosity of 76% and wall thickness about 0.4 mm, have UTS of about 76 MPa, while L-PBF gyroid with the same porosity and wall thickness of 0.5 mm demonstrates UTS of 60 MPa [35]. Sheet-based gyroid manufactured by L-PBF from Ti-6Al-4V with wall thickness of 0.25 mm and porosity varying from 78 to 87% can possess quasi-elastic gradient from 1.9 to 5 GPa, depending on the unit cell size [35]. The UTS for a gyroid with 6 × 6 × 6 mm unit cell and porosity of 87% was found to be equal to 24 MPa [35], equal to the results of the tensile testing of the WT gyroid (Table 3). However, the tensile strain at failure for EBM gyroid is approximately 10% while tensile failure strain of L-PBF gyroid with wall thickness of 0.25 mm ranges from 1.4% to 2.1%.

Achieving minimal thickness of the TPMS sheet-based lattices would yield many advantages: the structure would be lighter; it would be easier to remove entrapped powder; the cell size could be decreased, thereby allowing larger gradients. At the end of powder bed fusion processes, residual powder is always trapped in the final lattice. In the case of implants, if the powder will not be removed, inflammation and blockage of blood vessels may happen. To ensure efficiency of the dense porous structures in terms of bone cells’ ingrowth and mechanical properties, an adequate method for the trapped powder removal is still to be found. We assume that in the case if the gyroid sheet-based structures will be produced using Wafer Theme, the size of the unit cell can be decreased while maintaining the ability of effective powder removal from the structure. Moreover, the Wafer Theme can be implemented for manufacturing functionally graded porous structures with optimized pore size. Finally, such structures satisfy the requirements for medical implants to avoid stress-shielding effect.

In conclusion, TPMS WT structures present a few advantages (high strain tolerance in both tension and compression, relatively high quasi-elastic gradient, relatively low surface roughness), and a few caveats (systematic presence of holes at the saddle points of the structure), but prove to be well adapted to mimic human cortical bone. Therefore, such structures certainly merit more attention and further investigations as potential design geometries for load-tolerant lightweight structures used in biomedicine (implants) and technology.

## 5. Conclusions

TPMS gyroid sheet-based structures were, for the first time, successfully manufactured by EBM using Melt and Wafer Themes (MT and WT, respectively). To produce specimens in MT, a 0.2 mm thick model was used, while to produce specimens in WT, a zero-thickness model was used. It was initially assumed that in using proper input design parameters, corresponding EBM-manufactured MT and WT samples would have equivalent geometrical and mechanical properties. However, we observed that:

- The minimum mean wall thickness, which can be achieved using standard Melt Theme in ARCAM EBM A2 machine, is around 380 µm, while the minimum mean wall thickness with Wafer Theme is 250 µm.
- Despite the difference in thickness, quasi-elastic gradient and specific energy absorption at 50% strain are approximately the same. Thus, MT and WT structures behave identically at small strains up to 5% (in the elastic range) and have similar strain tolerance.
- WT gyroids exhibit through-hole defects in the surface sections perpendicular to the building direction. They supposedly appear in each horizontal saddle point because the areas of zero-thickness 3D model are not detected by slicing software and, therefore, are not processed by the beam. Through-holes connect two separate void regions which TPMS consist of, thus, enabling better fluid transport, tissue ingrowth and differentiation.
- FEA simulation revealed that the yielding of the metal initiates in the vertical areas located parallel to the load direction and continues in the diagonally oriented surfaces. The yielding process reaches horizontally aligned saddle points only at a later stage. Therefore, the through-holes influence the mechanical behavior only in the plastic region.
- Thus, the Wafer Theme EBM-manufacturing is a promising method for TPMS-based structures.

## Figures and Tables

**Table 1:** Specimen parameters.

Specimen Parameters	Melt Theme	Wafer Theme
Mass *m*, g	3.4 ± 0.1	2.3 ± 0.2
Volume *V*, cm^3^	3.4 ± 0.1	3.2 ± 0.1
Density ρ, g/cm^3^	1.07 ± 0.13	0.74 ± 0.15
ρ/ρ_0 (ρ0 = 4.43 g/cm3)_	0.24 ± 0.3	0.17 ± 0.2
Porosity p, %	76 ± 3	86 ± 5

**Table 2:** Summary of the results of the quantitative image analysis of XCT data. (Measurement errors cannot be estimated; the error intervals represent the variance of all the measured values).

Reconstructed Specimen Parameters	Melt Theme	Wafer Theme
Mean wall thickness, mm	0.38 ± 0.07	0.25 ± 0.06
Max. wall thickness, mm	0.56	0.44
Defect volume ratio, % (Micro-pores to bulk volume)	0.4	0.3

**Table 3:** Mechanical properties of the specimens.

Parameters	Compression		Tension	
	MT	WT	MT	WT
Porosity, %	76	85	76	85
Quasi-elastic gradient *E_qe_*, GPa	1.5 ± 0.1	1.5 ± 0.1	1.2 ± 0.1	1.2 ± 0.2
Compressive offset stress/Yield strength *σ_y_*, MPa	65 ± 1	30 ± 5	37 ± 5	5 ± 0.6
Yield strain, %	4.6	2.3	3.5	0.7
First maximum compressive strength/*σ_e_*, MPa	88 ± 2	40 ± 3		
UTS, MPa			76 ± 0.3	24 ± 0.6
Plateau stress *σ_p_*_l 20–40_, MPa	49 ± 2	15 ± 3	–	–
Energy absorption W_50_, MJ/m^3^	29 ± 0	11 ± 2	–	–
Specific energy absorption ψ (50%), J/g	27 ± 0	27 ± 1	–	–

**Table 4:** Ti-6Al-4V sheet-based gyroid properties.

Porosity, %	Wall Thickness, mm	*E_qe_,* GPa	Method	Reference
70	0.25	4.0	L-PBF	[4]
50	0.33	6.0	L-PBF	[4]
78	0.25	5.3	L-PBF	[35]
85	0.25	3.0	L-PBF	[35]
87	0.25	3.4	L-PBF	[35]
86	0.25 (WT)	1.5	EBM	Current
76	0.38 (MT)	1.5	EBM	Current

## Data Availability

The data presented in this study are available on request from the corresponding author. The data are not publicly available as they also form part of an ongoing study.
